# Using case-mixes to understand health resource utilization trajectories among older adults at high risk of falls who received baseline geriatrician-based Falls Prevention Clinic care

**DOI:** 10.1093/gerona/glag146

**Published:** 2026-06-02

**Authors:** Jennifer C Davis, Ryan S Falck, Chun Liang Hsu, Karim Khan, Patrick Chan, Cheyenne Ghag, Patrizio Jacova, Kenneth Madden, Larry Dian, Jordyn Rice, Naaz Parmar, Craig Mitton, Teresa Liu-Ambrose

**Affiliations:** Aging, Mobility, and Cognitive Neuroscience Laboratory, Department of Physical Therapy, University of British Columbia, Vancouver, British Columbia, Canada; Centre for Aging SMART, Vancouver Coastal Health Research Institute, Vancouver, British Columbia, Canada; Faculty of Management, University of British Columbia, Kelowna, British Columbia, Canada; Aging, Mobility, and Cognitive Neuroscience Laboratory, Department of Physical Therapy, University of British Columbia, Vancouver, British Columbia, Canada; Centre for Aging SMART, Vancouver Coastal Health Research Institute, Vancouver, British Columbia, Canada; Djavad Mowafaghian Centre for Brain Health, Vancouver Coastal Health Research Institute, Vancouver, British Columbia, Canada; Aging, Mobility, and Cognitive Neuroscience Laboratory, Department of Physical Therapy, University of British Columbia, Vancouver, British Columbia, Canada; Centre for Aging SMART, Vancouver Coastal Health Research Institute, Vancouver, British Columbia, Canada; Djavad Mowafaghian Centre for Brain Health, Vancouver Coastal Health Research Institute, Vancouver, British Columbia, Canada; Department of Rehabilitation Sciences, Faculty of Health and Social Sciences, The Hong Kong Polytechnic University, Hong Kong, China; Centre for Aging SMART, Vancouver Coastal Health Research Institute, Vancouver, British Columbia, Canada; Aging, Mobility, and Cognitive Neuroscience Laboratory, Department of Physical Therapy, University of British Columbia, Vancouver, British Columbia, Canada; Centre for Aging SMART, Vancouver Coastal Health Research Institute, Vancouver, British Columbia, Canada; Djavad Mowafaghian Centre for Brain Health, Vancouver Coastal Health Research Institute, Vancouver, British Columbia, Canada; Aging, Mobility, and Cognitive Neuroscience Laboratory, Department of Physical Therapy, University of British Columbia, Vancouver, British Columbia, Canada; Centre for Aging SMART, Vancouver Coastal Health Research Institute, Vancouver, British Columbia, Canada; Djavad Mowafaghian Centre for Brain Health, Vancouver Coastal Health Research Institute, Vancouver, British Columbia, Canada; Aging, Mobility, and Cognitive Neuroscience Laboratory, Department of Physical Therapy, University of British Columbia, Vancouver, British Columbia, Canada; Centre for Aging SMART, Vancouver Coastal Health Research Institute, Vancouver, British Columbia, Canada; Djavad Mowafaghian Centre for Brain Health, Vancouver Coastal Health Research Institute, Vancouver, British Columbia, Canada; Department of Medicine, Division of Geriatric Medicine, Faculty of Medicine, University of British Columbia, Vancouver, British Columbia, Canada; Department of Medicine, Division of Geriatric Medicine, Faculty of Medicine, University of British Columbia, Vancouver, British Columbia, Canada; Aging, Mobility, and Cognitive Neuroscience Laboratory, Department of Physical Therapy, University of British Columbia, Vancouver, British Columbia, Canada; Centre for Aging SMART, Vancouver Coastal Health Research Institute, Vancouver, British Columbia, Canada; Djavad Mowafaghian Centre for Brain Health, Vancouver Coastal Health Research Institute, Vancouver, British Columbia, Canada; Department of Medicine, Division of Geriatric Medicine, Faculty of Medicine, University of British Columbia, Vancouver, British Columbia, Canada; School of Population and Public Health, Faculty of Medicine, University of British Columbia, Vancouver, British Columbia, Canada; Aging, Mobility, and Cognitive Neuroscience Laboratory, Department of Physical Therapy, University of British Columbia, Vancouver, British Columbia, Canada; Centre for Aging SMART, Vancouver Coastal Health Research Institute, Vancouver, British Columbia, Canada; Djavad Mowafaghian Centre for Brain Health, Vancouver Coastal Health Research Institute, Vancouver, British Columbia, Canada; (Medical Sciences Section)

**Keywords:** Case-mix, Trajectory, Health resource utilization, Falls Prevention Clinic, Falls

## Abstract

**Background:**

We identified case-mixes among older adults at high risk of future falls, determined by longitudinal health resource utilization (HRU) cost trajectories, and examined baseline characteristics associated with these trajectories.

**Methods:**

This descriptive secondary cohort analysis included 343 community-dwelling older adults at high risk of falls who participated in a randomized controlled trial. All participants received evidence-based falls prevention care at the Falls Prevention Clinic. The primary outcome was total healthcare resource utilization costs collected prospectively over 12 months using self-report questionnaires and monthly cost diaries. Case-mixes were identified using 12-month trajectories and latent class growth modeling. Baseline characteristics examined included intervention group, age, biological sex, cognitive function, and physical function.

**Results:**

We identified 2 case-mixes. The “low-cost, stable” case-mix was characterized by low baseline HRU (∼$500 CAD) that remained stable over 12 months. The “low-cost, decreasing” case-mix had the lowest baseline HRU (<$500 CAD) and decreased over 12 months. Biological sex modified the trajectories. Specifically, males in the “low-cost, stable” case-mix demonstrated decreased HRU over 12 months, whereas female HRU remained unchanged. In the “low-cost, decreasing” case-mix, female HRU decreased at a greater rate than male HRU.

**Conclusions:**

Older adults at high risk of falls receiving Falls Prevention Clinic care demonstrated relatively low HRU characterized by stable and decreasing case-mixes. These findings provide descriptive insight into healthcare utilization patterns and may inform future evaluations of Falls Prevention Clinic models of care. These findings may assist future risk stratification and healthcare planning for older adults at elevated falls risk internationally.

The trial was registered at clinicaltrials.gov (NCT01029171; NCT00323596).

## Introduction

Falls among older adults are growing in prevalence and placing increasing demand and cost on the public health system.^1^^,^^2^ Medical costs attributable to fatal and nonfatal falls total $50.0 billion (2015 US dollars).^3^ Approximately 30% of adults aged 65 years and older experience a fall each year; of these falls, 20%-30% require medical attention, and approximately 5% result in a fracture, one-third of which are hip fractures.^4^ Consequences of falls are not homogeneous and include injuries of varied severity, loss of independence, and decreased mobility.^5^^,^^6^ These consequences further exacerbate individuals’ risk of future falls. Older adults at elevated risk of falls often experience greater frailty, multimorbidity, and functional decline, factors that may contribute to broader patterns of healthcare utilization costs beyond care directly linked to falls.

Research has made steady progress toward understanding risk factors for falls, ascertaining the efficacy and effectiveness of interventions to prevent falls, and identifying cost-effective approaches for falls prevention. Key risk factors for falls include impaired physiological function^7^^,^^8^ and cognitive impairment, defined as a Folstein’s Mini-Mental State Examination (MMSE) score < 24/30.^9^ A Cochrane review demonstrated high certainty evidence that exercise that included functional and balance components prevents falls,^10^ and a systematic review demonstrated that exercise delivered as a single intervention can prevent falls in community-dwelling older adults.^11^ Exercise is a cost-effective approach for falls prevention.^12^ Because older adults at high risk of falls often experience greater frailty, multimorbidity, and healthcare needs, preventing falls may influence broader patterns of healthcare utilization and associated costs over time. Yet, little is known about the longitudinal health care cost trajectories of individuals at high risk of falls.

Cost of falls studies have largely focused on providing global, country-specific, or fall categorization-specific estimates (i.e., fatal versus non-fatal, fall-related hospitalization, cost per faller or fall per person). Two systematic reviews that estimated the cost of falls in older age^13^ and the global cost of falls among community-dwelling older adults.^14^ One study demonstrated that national fall-related costs of falls accounted for 0.85% and 1.5% of the total health care expenditures, or 0.07% to 0.20% of the GDP of the United States (estimated range from 113 to 547 USD PPP per person).^13^ Older age groups, females, falls among those in hospital or long-term care facilities or falls resulting in fracture, demonstrated higher direct costs. Mean costs per fall person 2044 to 25 955, per fall 1059 to 10 913 and per fall-related hospitalization 5654 to 42 840 USD; these estimates were dependent on fall severity.^13^ A systematic review depicting the global cost of falls estimated that the mean cost of falls ranged from US $3476 per faller to US $10 749 per injurious fall and US $26 483 per fall requiring hospitalization.^14^

Case-mix classification provides an approach for summarizing heterogeneity in health care needs and resource utilization by grouping individuals with similar patterns of service use. In the context of falls, health resource utilization is largely examined using aggregate or cross-sectional measures, with limited attention to how patterns of use evolve following a fall event. Thus, it remains unclear whether distinct trajectories of health care utilization exist among older adults at high risk of falls, or whether a small number of utilization profiles account for a disproportionate share of costs. Applying a case-mix framework to longitudinal health resource utilization trajectories allows us to empirically identify and characterize these patterns, rather than assuming homogeneity among fallers. Identifying such case-mixes may provide insight into differing patterns of healthcare utilization among older adults with a recent fall history and high risk of future falls, including sustained high use, short-term increases in utilization, or relatively low ongoing healthcare contact. These classifications support health system planning, funding, and intervention targeting by capturing longitudinal patterns of health resource use rather than single care episodes.

Hence, we analyzed the health resource utilization cost trajectories among older adults at high risk of future falls participating in a randomized clinical trial^15^ over 12 months to: (1) identify distinct case-mixes in this cohort of older fallers as determined by their longitudinal health resource utilization cost trajectories; and (2) examine baseline characteristics associated with the health resource utilization case-mix groups identified in step 1. These case-mixes will first provide a better understanding of the health care usage patterns over a 12-month time horizon of adults at high risk of future falls. Examining baseline characteristics associated with distinct HRU trajectories may help identify subgroups of older fallers who experience differing patterns of healthcare use following a fall event. From a health services planning perspective, understanding predictors of longitudinal utilization patterns may inform future risk stratification, targeting of follow-up care, and development of tailored models of falls prevention delivery among older adults at high risk of falls.

## Methods

### Study design and setting

This study was a descriptive secondary cohort analysis using data collected from a randomized controlled trial of older adults at high risk of future falls.^15–18^ The present study used data from a 12-month randomized controlled trial involving community-dwelling older adults identified as being at elevated risk of falls. Participants were recruited through a specialized Falls Prevention Clinic and underwent multidisciplinary assessment prior to enrollment. Individuals were eligible if they were ambulatory older adults living in the community and met clinic referral criteria related to falls risk or mobility impairment. The clinic referral criteria included adults aged 65 years and older with a history of at least one fall in the previous 12 months, with priority given to individuals who had sustained a fall-related fracture. Eligible participants were ambulatory (with or without an assistive device), had preserved cognitive function (MMSE ≥24), and were expected to have a life expectancy greater than one year. Individuals with progressive neurological conditions, dementia, or Alzheimer’s disease were excluded. This study included 3 in-person measurement sessions at baseline, 6 months, and 12 months. Ethical approval was obtained from the University Clinical Research Ethics Board (H09-02370). All participants provided written informed consent.

### Recruitment

Participants were recruited from the Falls Prevention Clinic (www.fallsclinic.ca) from 2009 to 2017. All participants received a baseline evidence-based best practice care for falls prevention at the Falls Prevention Clinic. This included a falls risk factor assessment to identify individual risk factors for falling, followed by a comprehensive assessment by a geriatrician.^19^ The geriatrician performed a detailed one-hour medical examination, reviewing physical function, functional capacity, physical activity and exercise, nutrition, medication usage, alcohol and smoking habits, and potential hazards in the home. Briefly, the Falls Prevention Clinic model of care focuses is a multidisciplinary models of care that address the multifactorial causes of falls in older adults.^11^^-^^13^ These clinics typically involve comprehensive fall risk assessment, geriatrician consultation, medication review, and individualized exercise and education delivered by physiotherapists and allied health professionals.^14^ Existing evidence supports multifactorial falls prevention approaches for reducing falls and improving function among community-dwelling older adults. Prior work from the Vancouver Falls Prevention Clinic also demonstrated good feasibility and acceptability, with 65% clinic attendance and 69% adherence to provider recommendations among older adults with a history of falls.^12^ Most recently, data indicate that a Falls Prevention Clinic of care is a feasible, evidence-based approach that may reduce healthcare utilization improve outcomes among older adults at high risk of falls.^20^

### Eligibility

Recruited participants were aged 70 years and older and referred by a physician to the Falls Prevention Clinic secondary to a non-syncopal fall in the previous 12 months (i.e., all participants sustained a fall in the past 12 months and were at high risk for future falls). To be included, participants had to demonstrate a Physiological Profile Assessment© (PPA; Prince of Wales Medical Research Institute, Sydney, Australia)^21^ composite score of at least 1.0 *SD* above age-normative value, or a Timed Up and Go (TUG)^22^ test > 15 seconds, or experienced one additional non-syncopal fall in the previous 12 months.

Participants were excluded if they: (1) were previously diagnosed with or suspected (by the geriatrician) to have neurodegenerative disease or dementia; (2) had a stroke; or (3) had a history indicative of carotid sinus sensitivity (i.e., syncopal falls).

### Treatment group allocation

Participants were assigned to either the Otago Exercise Program or Usual Care (i.e., a comparator). Both groups received multidisciplinary care at the Falls Prevention Clinic. Additional details regarding trial procedures and intervention protocols have been reported previously.^15^

### Otago Exercise Program

Participants randomized to the intervention group received the Otago Exercise Program (OEP), an individualized home-based balance and strength retraining program previously described in detail.^15^^,^^23^^,^^24^ The OEP was delivered for 12 months, and all the physical therapy visits occurred in the first 6 months of the study.

### Usual care

Participants randomized to the control group received usual care that included a baseline assessment at the Falls Prevention Clinic with follow-up as requested by the geriatrician.

### Descriptive measures

We reported baseline demographic variables that included: age (years), education, comorbidities (using the Functional Comorbidity Index^25^ where a score range 0-18, 0 = best, indicating no comorbid illness), gait speed (meters per second), depression (using the 15-item Geriatric Depression Scale^26^^,^^27^; range 0-15, 0 = best; scores ≤5 are normal), the ability to live independently (using the Lawton and Brody^28^ Instrumental Activities of Daily Living Scale; range 0-8, 8 = best), and global cognitive function (using the MMSE^29^ and the Montreal Cognitive Assessment (MoCA)^30^; range 0-30 points for each measure, higher = better). Other descriptive measures included fall risk assessed by the Physiological Profile Assessment (PPA)^21^ (observed range −2 to 4, lower = better; 0-1 indicates mild risk, 1-2 indicates moderate risk, 2-3 indicates high risk, and 3 and above indicates marked risk). General balance and mobility were assessed using the Short Physical Performance Battery (SPPB)^31^ and the Timed Up and Go (TUG) test.^32^ The SPPB has a range of 0-12, with 12 indicating the best performance. An SPPB score of ≤9/12 predicts subsequent disability.^31^ A TUG score of ≥13.5 (range of 0-84) seconds indicates high fall risk; lower scores on the TUG indicate lower fall risk.^29^

### Health resource utilization

We tracked total healthcare resource utilization prospectively over 12 months using a self-report questionnaire and monthly cost diaries.^33^ HRU measures reflected total healthcare utilization captured during the study period and were not limited to fall-related healthcare use or injuries. The primary healthcare resource categories captured by the HRU and cost-diaries included visits with healthcare providers, hospital admissions or procedures, and diagnostic or laboratory investigations. Cost diary data were not validated against administrative health records, as linkage was outside the scope of the study. This study’s primary objective was to characterize relative patterns and trajectories of HRU over time rather than estimate absolute system-level costs. Because all participants completed the same prospective questionnaires and monthly cost diaries, we expected most reporting errors to be broadly similar across participants and therefore less likely to systematically bias comparisons between trajectory groups. Any nondifferential measurement error in self-reported utilization is unlikely to bias comparisons between trajectory groups.^33^^,^^34^ However, some differential reporting errors cannot be excluded. Unit costs for healthcare cost items were previously reported.^18^ A unit cost from the British Columbia Medical Serviced Plan (https://www2.gov.bc.ca/gov/content/health/practitioner-professional-resources/msp/physicians/payment-schedules/msc-payment-schedule) 2018 fee for service price list was assigned for each component of HRU. Hospital admission costs were based on a fully allocated cost model of a tertiary care hospital (a hospital that delivers a higher level of specialized care). Unit costs for specialized services (e.g., physiotherapy) were taken from the relevant British Columbia Association website. All costs were reported in 2019 Canadian Dollars. Discounting was not applied due to the 12-month time horizon.

### Adverse events

Three deaths occurred in the OEP group, and one death occurred in the usual care group; none of these was attributable to the intervention. These were recorded and reviewed by the Data Safety Monitoring Board.

### Statistical analysis

All analyses were conducted in R version 4.3.2. All HRU data were inflated by 11.10% as per bank of Canada in order to account for inflation to 2021 CAD levels. In order to improve model fit, we scaled HRU data such that each unit represented $1000 CAD. Further details on handling of missing data are described in the Supplementary File.

### Development of case-mix models for HRU over 12 months

Given that we were interested in developmental trajectory patterns of health resource utilization (HRU) over time, we used a group-based approach to identify distinct case-mixes of individual trajectories. Latent class growth modeling (LCGM) was selected to identify unobserved subgroups characterized by similar longitudinal trajectories of health resource utilization. Unlike clustering methods based on cross-sectional or aggregated measures, LCGM explicitly models change over time, enabling classification based on trajectory shape and level. Compared with growth mixture modeling, LCGM assumes within-class homogeneity, improving model stability and interpretability in large population-based administrative datasets. Latent transition analysis was not pursued because the study aimed to characterize sustained utilization patterns rather than transitions between discrete states. Overall, LCGM provides a parsimonious, trajectory-focused framework aligned with the study objective of describing longitudinal health resource use patterns.^35^^,^^36^ We applied latent class growth modeling based on the Nagin model using a group-based trajectory model to consider subgroups (i.e., case-mixes).^36^ Case-mixes were empirically derived, data-driven groups identified using latent class growth modeling, representing individuals who followed similar longitudinal trajectories of health resource utilization over time. We used the *lcmm* package in R to estimate case mixes.^37^ This package provides estimation functions for latent class mixed models, which are estimated using maximum likelihood. Latent class mixed models assume that the population is heterogeneous and composed of *G* latent classes of subjects characterized by *G* mean profiles of trajectories. Each subject belongs to only one latent class.

Our model development and results can be found in a GitHub repository (https://github.com/ryanfalck/AS_Case_Mix_Trajectories). First, we explored how to fit HRU data using 3 link functions were examined: (1) a linear link function; (2) a spline link function using 3 equidistant splines; and (3) a spline function using 3 quantiles located at 25th, 50th, and 75th percentiles. Each link function was tested wherein time was treated as either a first-order, second-order (i.e., quadratic), or third-order (i.e., cubic) variable. Nine models were compared in total. We did not include covariates in any model. We then compared model fit according to AIC, BIC, and the log-likelihood function (see Supplemental Table S1). The 5 best-fitting models were further investigated.

For the 5 models selected, we then examined the number of classes that best fit the data. We compared model fit from 1 to 6 classes according to AIC, BIC, log-likelihood, and entropy. We selected as our final model the most accurate, parsimonious, and easily interpretable model, which we then used to predict HRU trajectories for each case-mix. In addition, we estimated the probability of case-mix membership and posterior probability of case-mix membership (i.e., the collective measurement of each participant’s probability). Values of the average posterior probability of membership were assessed within each case-mix; average posterior probability of >0.7 indicates adequate internal reliability.^38^ Our final case-mix model included 4 distinct classes. Two classes comprising *n* = 3 and *n* = 9 participants were identified in higher-order solutions. These classes showed extremely low sample sizes, limiting their external validity and were therefore excluded from the final model.

Given that economic data are usually skewed (i.e., participants either substantially utilize health resources or do not at all), we examined, as a sensitivity analysis, whether normalizing or log-transforming HRU data improved model fit. Two separate models were developed using: (1) HRU values, which were normalized using a min-max normalization procedure; and (2) log-transformed HRU values. Min-max normalization was conducted using the following equation:


$$\frac{\mathrm{HRUvalue}-\mathrm{HRUmin}}{\mathrm{HRUmax}-\mathrm{HRUmin}}$$

Wherein *HRUvalue* represented the given HRU amount, *HRUmin* was the absolute minimum HRU value (i.e., $0), and *HRUmax* was the maximum HRU amount ($50 314).

### Investigation of baseline predictors of case-mix trajectory

After selecting the best-fitting model, we then examined whether the case-mix trajectories were moderated by the baseline predictors of (1) intervention group, (2) age, (3) biological sex, (4) cognitive function, or (5) physical function. We conducted 5 separate interaction latent class mixed models wherein HRU total cost was the outcome of interest, and time and the baseline predictor of interest, as well as their interaction, were treated as fixed effects. Fixed effects were calculated for each model; we used Wald statistics in order to estimate interaction effects for each case-mix trajectory.

## Results

Table 1 reports descriptive characteristics for the full sample as well as the descriptive statistics for each of the 4 case mixes identified. Mean age was 82 years (*SD* = 6 years), and 67.5% were female. The Mean baseline Montreal Cognitive Assessment (MoCA) score was 23.15 (*SD* = 3.36), and 47.4% of participants had a university degree. Participants reported an average of 3 falls (*SD* = 2) in the 12 months prior to starting the study. Case-mix 1 and case-mix 3 included only 3 and 9 participants, respectively; we did not include these groups in our final analyses.

**Table 1:** Baseline characteristics of participants by case-mix trajectory for health resource utilization.

	Full sample (*N* = 343)	Case-mix 1 (*N* = 3)	Case-mix 2 (*N* = 245)	Case-mix 3 (*N* = 9)	Case-mix 4 (*N* = 86)	*p*
**Assigned to OEP *n*, %**	172, 50.1	2, 66.7	122, 49.8	3, 33.3	45, 52.3	.678
**Age**	81.58 (6.09)	79.67 (6.11)	81.32 (6.15)	85.22 (4.87)	81.99 (5.96)	.227
**Males *n*, %**	115, 33.5	3, 100	82, 33.5	5, 55.6	25, 29.1	.034
**Body mass index (kg/m^2^)**	27.22 (6.41)	29.51 (3.18)	26.98 (4.96)	24.99 (3.27)	28.05 (9.61)	.377
**Geriatric depression scale-15**	2.87 (2.52)	0.67 (0.58)	2.80 (2.42)	2.89 (2.26)	3.15 (2.83)	.316
**Functional comorbidity index**	3.78 (1.99)	3.33 (1.53)	3.73 (2.06)	3.78 (1.64)	3.95 (1.86)	.813
**Instrumental activities of daily living**	7.30 (1.17)	6.33 (1.53)	7.33 (1.16)	6.88 (0.99)	7.30 (1.20)	.351
**SPPB**	7.87 (2.25)	5.67 (2.52)	8.02 (2.24)	7.89 (3.02)	7.51 (2.17)	.107
**Mini mental state exam**	27.76 (1.63)	26.00 (1.00)	27.82 (1.60)	27.67 (1.73)	27.66 (1.71)	.250
**Montreal cognitive assessment**	23.15 (3.36)	23.00 (3.46)	23.20 (3.35)	24.33 (3.43)	22.88 (3.39)	.632

### Case-mix models for HRU over 12 months

**Figure 1:**
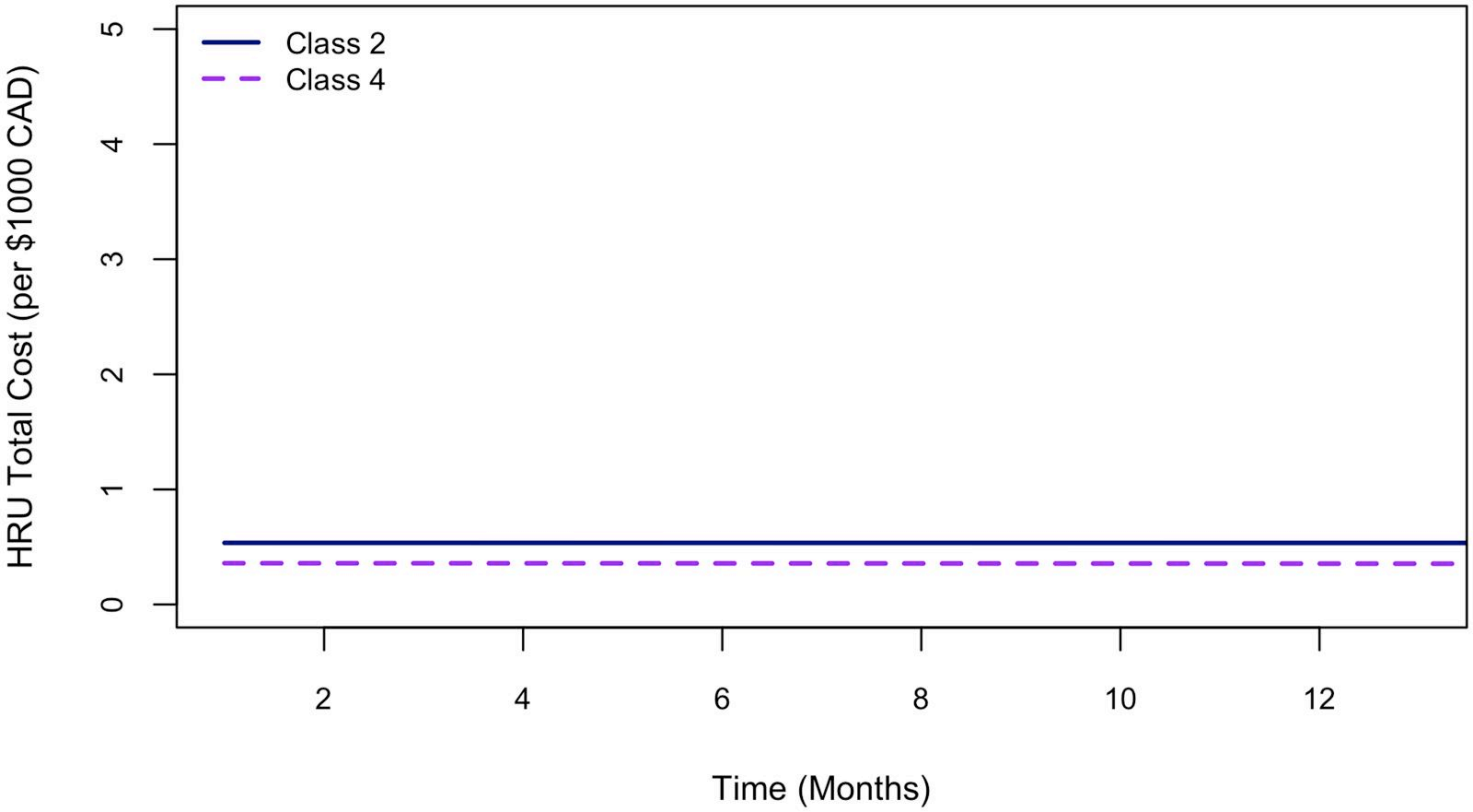
Case-mix trajectories for health resource utilization across 12 months.

We describe model fit statistics for the initial models investigated in Table 2. For our final model, we selected a linear model with a spline function using 3 quantiles located at the 25th, 50th, and 75th percentiles and 4 classes. Case-mix 1 (*n* = 3) and case-mix 3 (*n* = 9) were excluded from our analyses, given their small sample sizes. Figure 1 describes the two analyzed case-mixes. These cases-mixes were defined visually as follows:

**Case-mix 2 “low-cost, stable” (*n* = 245)**, included 71% of participants and was characterized by a modest HRU utilization at baseline (∼$500 CAD), which was consistent across 12 months.

**Case-mix 4 “lowest-cost, decreasing” (*n* = 86)**, included 25% of participants and was characterized by the lowest HRU utilization at baseline (i.e., <$500 CAD) and decreased over 12 months.

**Table 2:** Model fit statistics.

Model type	Link function	Classes	Log likelihood	AIC	BIC	Entropy	% in class 1	% in class 2	% in class 3	% in class 4	% in class 5	% in class 6
**Linear**	Spline	4	−1134.47	2304.95	2374.03	0.79	0.87	71.43	2.62	25.07		
**Quadratic**	Spline	3	−1062.68	2167.36	2247.95	0.99	1.46	97.67	0.87			
**Quadratic**	Spline	4	−1079.45	2208.89	2304.83	0.72	1.46	70.85	23.91	3.79		
**Quadratic**	Spline	5	−1058.75	2175.51	2286.8	0.67	1.46	1.46	94.17	0.87	2.04	
**Quadratic**	Spline	6	−1048.67	2163.34	2289.99	0.84	1.17	1.46	67.06	27.11	0.87	2.33

### Moderating effects of predictors of case-mix trajectories

**Figure 2:**
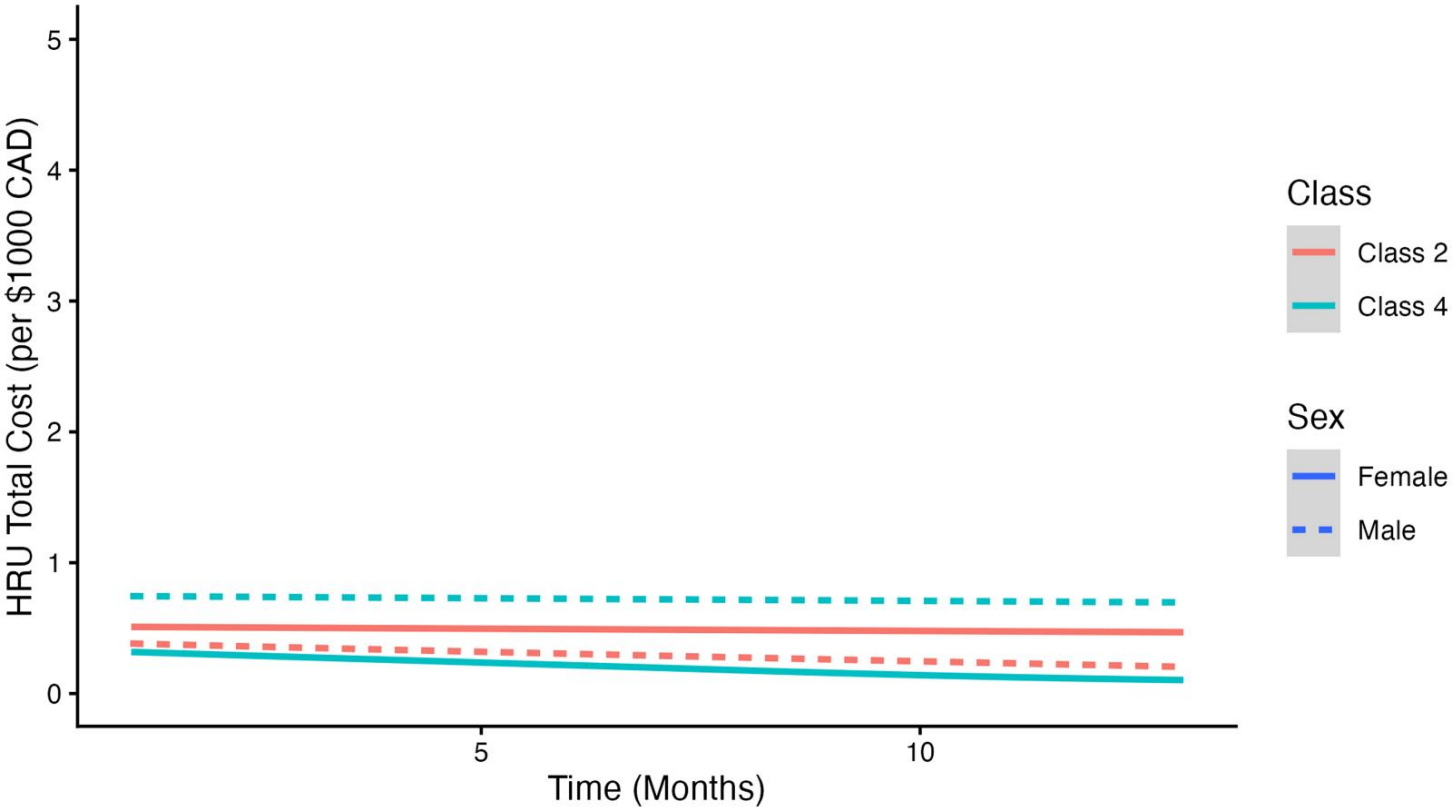
Moderation of case-mix trajectories by biological sex.

Moderating effects for each predictor of case-mix trajectory are described in Table 3. Biological sex moderated the trajectories for **Case-mixes** 2 and 4. Specifically, males in **Case-mix** 2 decreased HRU over 12 months, while female HRU did not appear to substantially change. For **Case-mix** 4, female HRU decreased at a greater rate over 12 months than for males. These moderating effects are described in Figure 2. We did not observe any additional moderating effects on case-mix trajectories.

**Table 3:** Baseline moderators of case-mix trajectories for health resource utilization.

		Group			Time			Group × Time		
Moderator	Class	β ± *SE*	Wald statistic	*p*	β ± *SE*	Wald statistic	*p*	β ± *SE*	Wald statistic	*p*
**Intervention group**	Class 1	–	–	–	–	–	–	–	–	–
	Class 2	0.07 ± 0.11	0.65	.519	0.01 ± 0.01	0.35	.730	−0.01 ± 0.01	−1.05	.293
	Class 3	–	–	–	–	–	–	–	–	–
	Class 4	−0.99 ± 0.65	−1.52	.128	0.23 ± 0.05	4.77	<.001	0.09 ± 0.09	0.97	.332
**Age**	Class 1	–	–	–	–	–	–	–	–	–
	Class 2	0.08 ± 0.12	0.67	.501	0.01 ± 0.01	0.23	.815	−0.01 ± 0.01	−1.14	.256
	Class 3	–	–	–	–	–	–	–	–	–
	Class 4	−0.03 ± 0.17	−0.17	.867	−0.08 ± 0.02	−4.93	<.001	0.01 ± 0.02	0.35	.723
**Biological sex**	Class 1	–	–	–	–	–	–	–	–	–
	Class 2	0.62 ± 0.16	3.87	<.001	−0.10 ± 0.03	−3.83	<.001	0.05 ± 0.02	2.69	.007
	Class 3	–	–	–	–	–	–	–	–	–
	Class 4	0.62 ± 0.16	3.87	.005	0.26 ± 0.11	2.37	.018	−0.26 ± 0.07	3.64	<.001
**Mild cognitive Impairment**	Class 1	–	–	–	–	–	–	–	–	–
	Class 2	−0.13 ± 0.11	−1.15	.250	−0.01 ± 0.01	−1.45	.148	0.02 ± 0.01	1.37	.169
	–	–	–	–	–	–	–	–	–	–
	Class 4	−0.30 ± 0.54	−0.59	.552	0.17 ± 0.04	4.62	<.001	0.06 ± 0.06	0.93	.351
**Frailty status**	Class 1	–	–	–	–	–	–	–	–	–
	Class 2	0.06 ± 0.16	0.38	.706	−0.04 ± 0.02	−2.64	.008	−0.03 ± 0.02	−1.61	.107
	Class 3	–	–	–	–	–	–	–	–	–
	Class 4	−0.03 ± 0.48	−0.07	.948	0.20 ± 0.04	4.92	<.001	−0.02 ± 0.06	0.29	.771

## Discussion

A novel element of this study was to identify that among individuals at high risk of falls who received a comprehensive medical assessment at a geriatrician-based Falls Prevention Clinic, low-cost health resource use was observed over the following 12-months. Two case-mixes identified demonstrated low-cost, stable or decreasing health resource utilization over 12 months. These findings of low-cost health resource utilization contrast with current literature. The cost of falls varies depending on injury severity and recovery needs. The average cost of falls with injury ranges from $CAD10K-15K.^39^ Thus, the costs of falls can be substantial among individuals at high-risk of falling. The relatively low HRU trajectories observed in this cohort provide descriptive insight into the overall low healthcare utilization patterns among older adults receiving care through the Falls Prevention Clinic model of care. Because we lack a “do-nothing” comparator, these findings should not be interpreted directly as evidence of cost savings.

We demonstrated that individuals who are at high risk of falls had two primary patterns of low-cost health resource utilization (stable and decreasing). The case mixes were moderated primarily by sex. Males in the “low-cost, stable” case-mix decreased HRU over 12 months, while female HRU did not change. For the “low-cost, decreasing” case-mix, female HRU decreased at a greater rate than males. These findings may reflect differing healthcare utilization and care-seeking patterns among older males and females following falls.^4^^,^^40^^,^^41^ For example, women have demonstrated higher health-seeking behaviors for different modalities of health care such as screening (i.e., preventative), rehabilitation services (i.e., outpatient), and emergency department visits (i.e., acute).^4^^,^^40^^,^^41^ Men have demonstrated delayed care-seeking tendencies for preventative, rehabilitation, and outpatient care, which is associated with worse outcomes.^42^ These findings build on prior literature demonstrating sex differences in rehabilitation use, preventative care engagement, and emergency department utilization. While our analyses examined total HRU rather than fall-attributable utilization specifically, these trajectory differences suggest that sex may be an important consideration in understanding patterns of healthcare use among older adults at high risk of falls.

Notably, intervention group allocation was not associated with differing HRU trajectories over time. Similarly, several other baseline characteristics were not associated with trajectory group membership, underscoring the complexity and multidimensional nature of healthcare utilization among older adults at high risk of falls. HRU in older populations is often influenced by many factors including medical complexity, frailty, multimorbidity, social support, functional decline, and healthcare access.^43^ Our findings suggest that healthcare utilization trajectories may remain difficult to predict even within clinically high-risk populations, particularly when broader system-level and behavioral factors contribute to patterns of care use over time.

Although falls are associated with increased health care costs, case-mixes based on health resource utilization patterns over time remain unknown. In this study, the majority of participants were grouped into the low-cost stable case-mix. These data suggest that a number of fallers exhibit stable patterns of health resource utilization after a comprehensive and specialized medical assessment. There are 5 benefits of fallers demonstrating these stable patterns. First, it allows for predictable budgeting and cost management, and this aids in forecasting healthcare expenditures to facilitate better financial planning and resource allocation. Predictability is an asset for efficiently managing healthcare budgets.^44^ Second, identifying stable and low-cost case-mixes can provide benchmarks and best practices for healthcare delivery, such that these models of care can be analyzed for their effectiveness and efficiency to improve outcomes and reduce costs to the system.^45^ Third, understanding characteristics of case-mixes that are low-cost and stable resource utilization users facilitates better population health management.^46^ Fourth, it can enable improved risk stratification and targeted interventions, and resource allocation. Finally, a possible benefit of low-cost stable health resource utilization case-mixes is that they contribute to improved financial performance by lowering the utilization of unnecessary services and focusing on quality outcomes.^47^ This aligns with the broader goals of value-based care, like that provided by the Falls Prevention Clinic, aimed to enhance patient outcomes while controlling health resource utilization costs.

### Limitations

Every participant received the Falls Prevention Clinic intervention, and thus, health resource utilization rates may be lower due to the care received. Thus, we were not able to determine the specific cost impacts of the Falls Prevention Clinic. Second, two case-mix groups had fewer than 3% of the study participants (*n* = 3 and *n* = 9). We excluded these two case-mix groups from our analysis and discussion since their small sample sizes suggest their parameter estimates may be unstable and less likely to be replicated. Notably, there were no important differences in demographics, physical function, or cognitive function between these case-mix groups and the two larger case-mix groups, suggesting that the larger case-mix groups are more interpretable, and likely more reliable and reproducible solutions. Given the small sample, statistical tests of moderation and inferences for HRU trajectories were not conducted. Third, all participants included sustained at least one fall in the previous 12 months at baseline and were in the high-risk for falls category. Thus, our findings cannot be generalized to the general population, non-fallers, or other levels of falls risk such as low or moderate falls risk. Health resource utilization was measured monthly via self-report. We note that this subject to recall bias, and it is possible that costs for health resource utilization were under-reported.^33^ It is possible that reporting accuracy differed across participants based on factors such as cognitive or health status. We did not define HRU by pre-fall versus post-fall care, given that HRU measures captured total healthcare utilization over the study period and were not restricted to fall-related care. Because total HRU rather than fall-attributable HRU was examined, observed trajectory differences should not be interpreted as direct evidence of differential fall-related healthcare needs or effectiveness of sex-specific falls prevention interventions. Because participants had experienced falls prior to study inclusion, observed HRU trajectories may partially reflect healthcare use associated with prior fall events as well as broader healthcare needs linked to elevated future fall risk. This study did not include a comparator or usual care group. Hence, we were unable to determine whether the observed HRU trajectories reflect reduced healthcare utilization or cost savings attributable to the Falls Prevention Clinic model of care.

## Conclusion

We describe health resource utilization patterns over 12 months among older adults at high risk of future falls who received a comprehensive baseline assessment at a geriatrician-led Falls Prevention Clinic. Participants demonstrated relatively low and stable health resource use during the year following their assessment. These findings provide a descriptive characterization of post-assessment utilization trajectories within this clinical population, highlighting heterogeneity in resource use among older adults referred for fall risk evaluation and who received a multidisciplinary falls geriatrician-based falls prevention service. While the absence of a comparator group precludes causal inference regarding the effect of clinic-based care on costs, the observed patterns generate hypotheses for future comparative and economic evaluations examining the potential role of a multidisciplinary falls prevention service model of care in lowering downstream health system use.

## Supplementary Material

glag146_Supplementary_Data

## Data Availability

The data from this manuscript cannot be shared publicly due to privacy, ethical, and institutional restrictions, as the dataset contains potentially identifiable participant information. Data may be available from the corresponding author upon reasonable request and with permission from the relevant institutional ethics board(s), subject to applicable data sharing agreements and approvals.
