# Supplementary material for: Using case-mixes to understand health resource utilization trajectories among older adults at high risk of falls who received baseline geriatrician-based Falls Prevention Clinic care
Source: J Gerontol A Biol Sci Med Sci. 2026 Jun 2;81(7):glag146. doi: 10.1093/gerona/glag146 (PMC13290473; doi:10.1093/gerona/glag146)
Supplement: glag146_Supplementary_Data [file glag146_supplementary_data.zip › 18-Jun-2026_043322_Supplemental_File_1_June_15_2026.pdf]

Supplemental File 1

Methods

Missing data and uncertainty

We imputed missing health related quality of life and healthcare resource use data. Missing data from each follow-up period for each participant were determined separately for both cost and effectiveness outcomes. We imputed missing EQ-5D-3L, SF-6D, and healthcare resource use values at each time point. For each missing value, we generated five possible values using multiple linear regression. Covariates included age, trial group allocation, baseline utility score, and the weight and value of the missing variable in the preceding period. The final imputed value was the mean value from the five data sets created. In our sensitivity analysis we explored uncertainty surrounding the point estimate of the incremental cost-effectiveness ratios using deterministic assumptions and bootstrap methods. For example, we restricted our data to a complete case analysis, thus including only participants for whom we had complete cost and effectiveness data to eliminate uncertainty caused by missing data. We analyzed total healthcare resource utilization and fall related healthcare resource utilization costs separately in our sensitivity analysis. As mentioned above, we applied multiple imputation, bootstrapped confidence interval estimation, adjustment for imbalances in baseline utility and bootstrapped estimates of the incremental cost effectiveness and cost utility ratios.

Results

Costs

Complete healthcare resource utilization data were provided by 171 (99%) participants over the 12-month time horizon.

Health Outcomes

Complete data for the EQ-5D-3L were provided at all time points (3-month, 6-month, 9-month, 12-month) for 141 (82%) participants in the exercise group, and 149 (87%) participants in the usual care group. In the exercise group, at 3-months, 168 (98%) had complete EQ-5D-3L data, at 6-months 156 (91%) had complete EQ-5D-3L data, at 9-months 147 (85%) had complete EQ-5D-3L data, and at 12-months 141 (82%) had complete EQ-5D-3L data. In the "usual care", at 3-months, 168 (98%) had complete EQ-5D-3L data, at 6-months 159 (92%) had complete EQ-5D-3L data, at 9-months 151 (88%) had complete EQ-5D-3L data, and at 12-months 149 (87%) had complete EQ-5D-3L data. Complete data for the SF-6D were provided at all time points (3-months, 6-months, 9-months, 12-months) for 140 (81%) participants in the exercise group, and 144 (84%) participants in the usual care group. In the exercise group, at 3-months, 172 (100%) had complete SF-6D data, at 6-months 157 (91%) had complete SF-6D data, at 9-months 144 (84%) had complete SF-6D data, and at 12-months 140 (81%) had complete SF-6D data. In the "usual care", at 3-months 172 (100%) had complete SF-6D data, at 6-months 154 (90%) had complete SF-6D data, at 9-months 149 (87%) had complete SF-6D data, and at 12-months 144 (84%) had complete SF-6D data.

Supplementary Figure 1

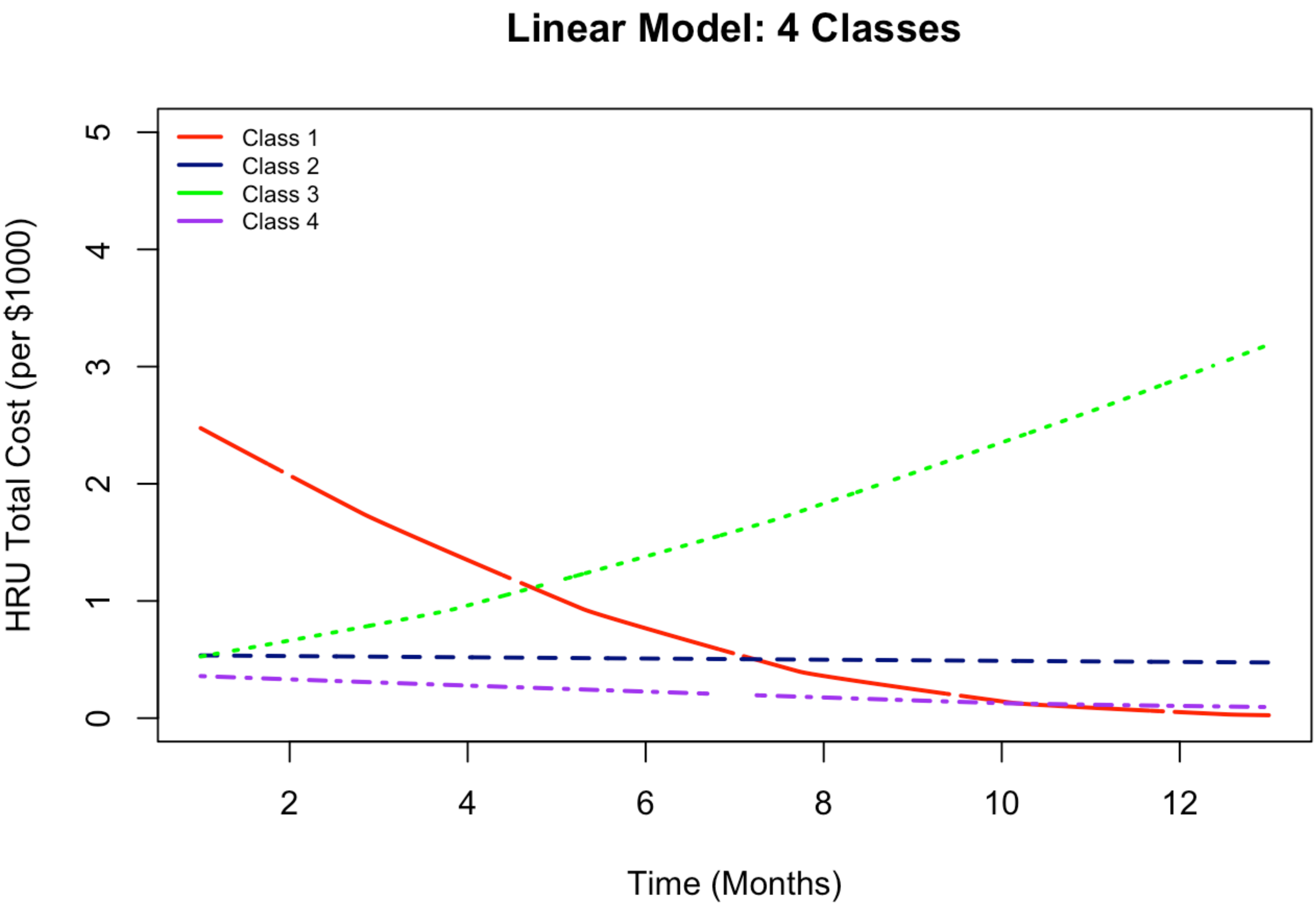

Supplementary Table S1. Model Fit Indices for Link Functions

Model	Parameter Estimates	Log Likelihood	AIC	BIC
Linear				
1st Order	6	-8665.68	17343.36	17366.39
2nd Order	10	-8626.89	17273.77	17312.15
3rd Order	15	-12789.19	25608.39	25665.95
Equidistant Spline				
1st Order	9	-4992.355	10002.71	10037.25
2nd Order	13	-4980.61	9987.22	10037.11
3rd Order	18	-11957.57	23951.13	24020.21
Quantile Spline				
1st Order	9	-1165.17	2348.33	2382.87
2nd Order	13	-1150.68	2327.36	2377.25
3rd Order	18	-7320.98	14677.96	14747.04

Supplementary Table S2. Model fit indices for best fitting link functions

Model	Parameter Estimates	Log Likelihood	AIC	BIC	Entropy	% Class 1	% Class 2	% Class 3	% Class 4	% Class 5	% Class 6
Linear 1st Order											
1 Class	9	-1165.17	2348.33	2382.87	1	100					
2 Classes	12	-1154.39	2332.77	2378.83	0.92	2.92	97.08				
3 Classes	15	-1154.39	2338.77	2396.34	0.48	3.21	96.79	0			
4 Classes	18	-1134.47	2304.95	2374.03	0.79	0.87	71.43	2.62	25.07		
5 Classes	21	-1134.46	2310.92	2391.51	0.59	0.87	29.45	67.06	2.62	0	
6 Classes	24	-1129.36	2306.72	2398.83	0.67	2.33	64.72	2.33	0.87	29.74	0
Quantile Spline 2nd Order											
1 Class	13	-1150.68	2327.36	2377.25	1	100					
2 Classes	17	-1088.55	2211.11	2276.35	0.99	1.46	98.54				
3 Classes	21	-1062.68	2167.36	2247.95	0.99	1.46	97.67	0.87			
4 Classes	25	-1079.45	2208.89	2304.83	0.72	1.46	70.85	23.91	3.79		
5 Classes	29	-1058.75	2175.51	2286.8	0.67	1.46	1.46	94.17	0.87	2.04	
6 Classes	33	-1048.67	2163.34	2289.99	0.84	1.17	1.46	67.06	27.11	0.87	2.33
